# Advances in Novel Protein Acylation in Urothelial Carcinoma: Research Progress and Biomarker Potential

**DOI:** 10.32604/or.2026.084336

**Published:** 2026-10-28

**Authors:** Xiyue Dong, Jiankun Zhang, Meixi Liu, Ruiqiang Sun, Yuanshan Cui

**Affiliations:** 1 School of Clinical Medicine, Shandong Second Medical University, Weifang, China; 2 Department of Urology, Weifang People’s Hospital, Weifang, China; 3 Weifang No. 1 Middle School, Weifang, China

**Keywords:** Protein acylation, lactylation, succinylation, crotonylation, palmitoylation, urothelial carcinoma (UC)

## Abstract

Protein acylation serves as an important molecular bridge between metabolic status and cellular function. It is becoming a key entry point for understanding tumor initiation, progression, and treatment resistance. In urothelial carcinoma (UC), metabolic reprogramming may alter the availability of acyl donors, including lactyl-CoA (coenzyme A), succinyl-CoA, crotonyl-CoA, and palmitoyl-CoA. This can affect acylation on both histone and non-histone proteins. This review focuses on four metabolism-related acylation modifications: lactylation, succinylation, crotonylation, and palmitoylation. Itummarizes their regulatory mechanisms, biological functions, and recent research progress in cancer. It further discusses their potential roles in UC progression, immune remodeling, inflammation regulation, treatment resistance, and biomarker development. Current evidence suggests that metabolism-dependent acylation modifications may promote malignant progression of UC by coordinating tumor metabolism, epigenetic regulation, and oncogenic signaling pathways. Therefore, this review aims to summarize recent advances in novel protein acylation modifications in UC, clarify their dynamic regulatory networks, and discuss their potential value in disease classification, prognostic assessment, therapeutic target identification, and personalized treatment strategies.

## 1 Introduction

Urothelial carcinoma (UC) is the most common histological type of urinary tract malignancy and can arise in the bladder, upper urinary tract, and urethra. According to the Global Cancer Statistics 2024, approximately 635,000 new cases of bladder cancer and 228,000 associated deaths occurred worldwide in 2024, ranking bladder cancer as the eighth most commonly diagnosed cancer globally [1]. Although treatment strategies have improved over the past two decades, the overall mortality of UC has shown limited improvement [2,3].

From a clinical perspective, UC is marked by high recurrence and a substantial risk of metastasis, which complicate disease management. Although immune checkpoint inhibitors (ICIs) have expanded treatment options, their efficacy remains constrained by the patient’s immune status and the tumor microenvironment (TME) [4,5]. Given the broad molecular subtypes and marked intratumoral heterogeneity of UC, some subsets respond suboptimally to immunotherapy, underscoring the need to identify new therapeutic targets and robust biomarkers [6].

Post-translational modifications (PTMs) are important dynamic regulatory processes in cells. They can rapidly convert metabolic and microenvironmental signals into changes in protein structure, localization, stability, activity, and interaction networks. These modifications play key roles in tumor initiation, progression, and drug resistance.

In addition to common phosphorylation and acetylation, the development of high-resolution mass spectrometry has led to the discovery of other novel protein acylation modifications. These modifications are closely linked to central carbon metabolism, amino acid metabolism, mitochondrial metabolism, and lipid metabolism, because their acyl donors are derived from metabolites such as lactate, succinyl-CoA, crotonyl-CoA, and palmitoyl-CoA [7]. In cancer, these metabolite-regulated acylation processes can serve as direct molecular links between metabolic reprogramming and malignant phenotypes.

This review focuses on lactylation, succinylation, crotonylation, and palmitoylation because they represent four metabolism-related but functionally distinct acylation modifications. These modifications are linked to lactate, succinyl-CoA, crotonyl-CoA, and palmitoyl-CoA, and may affect chromatin regulation, mitochondrial metabolism, membrane signaling, immune remodeling, and treatment response [8,9,10,11,12]. However, not all of these mechanisms have been directly demonstrated in UC. Therefore, this review prioritizes UC-specific evidence and uses findings from other cancer models only as hypothesis-generating evidence.

Novel protein acylation modifications are becoming increasingly important in UCresearch. These modifications may reshape UC biology at multiple regulatory levels, including chromatin-related transcriptional control, mitochondrial metabolic regulation, non-histone protein activity, membrane-associated oncogenic signaling, immune escape, and treatment tolerance.

Under the framework of “metabolic reprogramming-driven acylation regulation”, this review aims to summarize recent advances in novel protein acylation in UC, clarify the regulatory networks linking metabolic reprogramming with acylation modification, and discuss their potential value as biomarkers and therapeutic targets. By integrating current evidence and recent research progress, this review may help improve our understanding of the complex regulatory network in UC. Throughout this review, findings obtained from non-UC models are not presented as direct evidence for UC. Instead, they are used only as hypothesis-generating evidence to guide future UC-specific validation. If these basic findings can be translated into clinical practice, they may improve early diagnosis, therapeutic efficacy, and personalized treatment in UC.

## 2 Search Strategy

This article is a narrative review rather than a systematic review or meta-analysis. We searched PubMed/MEDLINE, Embase, Web of Science Core Collection, and Scopus for English-language studies from database inception to February 2026. Search terms included “urothelial carcinoma”, “bladder cancer”, “upper tract urothelial carcinoma”, “protein acylation”, “lactylation”, “succinylation”, “crotonylation”, “Kcr”, “palmitoylation”, “post-translational modification”, “tumor microenvironment”, “immune evasion”, “metabolism”, “biomarker”, “prognosis”, “therapy”, and “resistance”.

We prioritized primary studies that examined lactylation, succinylation, crotonylation, palmitoylation, or their regulatory enzymes/substrates in UC tissues, UC cell lines, animal models, patient cohorts, or public UC datasets. When UC-specific evidence was unavailable, mechanistically informative studies from other cancer types were included and labelled as extrapolated rather than direct UC evidence.

For each representative study, we recorded the cancer type or model system, the acylation modification or pathway, the main biological finding, and whether the evidence was UC-specific. This evidence map is summarized in Table 1. A separate translational table summarizes candidate biomarkers or therapeutic axes, the cancer type in which evidence is available, clinical associations, and validation status (Table 2).

The final reference set was curated after removing duplicated, malformed, weakly related, and low-relevance citations. Preference was given to PubMed-indexed primary studies, high-impact mechanistic papers, and focused reviews that directly support the text. Bibliometric articles, non-cancer disease papers, and non-UC studies were retained only when they supplied a necessary mechanistic principle that could not be supported by stronger UC-specific work.

**Table 1:** Representative evidence for acylation-related mechanisms and their relevance to urothelial carcinoma (UC).

Study	Cancer Type/Model	Modification or Pathway	Key Finding
**UC-specific evidence**			
Xie et al. (2023) [13]	Bladder cancer tissues and models	circXRN2-*LATS1*-Hippo axis; H3K18 lactylation	circXRN2 activated Hippo signaling by stabilizing LATS1, reduced lactate production and H3K18 lactylation, and suppressed *LCN2*-mediated malignant progression.
Wang et al. (2025) [14]	Bladder cancer tissues and models	*HNRNPA1*-K350 lactylation; *PKM2* splicing	HNRNPA1 lactylation promoted PKM2 splicing and glycolytic reprogramming, supporting bladder cancer malignant phenotypes.
Deng et al. (2025) [15]	Bladder cancer tissues, macrophage models, animal models	H3K18 lactylation; PRKN-mediated mitophagy	H3K18 lactylation promoted M2-like macrophage polarization and was associated with poor prognosis.
Wu et al. (2025) [16]	Bladder cancer cells, tissues, TCGA, immunotherapy cohort	MPP1; USP12/CCL5 axis	Higher MPP1 expression enhanced CD8^+^ T-cell recruitment, suppressed immune evasion, and correlated with improved prognosis and immunotherapy response.
Li et al. (2024) [17]	Bladder cancer models	*ZDHHC9*-mediated BiP/GRP78 S-palmitoylation	ZDHHC9-mediated BiP/GRP78 palmitoylation inhibited unfolded protein response and promoted bladder cancer progression.
**Extrapolated from other cancers**			
Zhu et al. (2025) [18]	Glioma and immune models	*ACSS2-KAT2A* lactylation axis	*ACSS2* coupled *KAT2A* to histone lactylation and immune evasion.
Li et al. (2020) [19]	Gastric cancer tissues and cells	*LDHA* K222 succinylation	LDHA succinylation stabilized *LDHA* and promoted tumor growth and metastasis.
Yan et al. (2025) [20]	Breast and ovarian cancer models	B7-H4 palmitoylation; *ZDHHC3*	B7-H4 palmitoylation maintained immunosuppressive stability; inhibiting palmitoylation enhanced T-cell activity.

Abb: H3K18, histone H3 lysine 18; H3K18la, histone H3 lysine 18 lactylation; TCGA, The Cancer Genome Atlas; CD8, cluster of differentiation 8; BiP/GRP78, binding immunoglobulin protein/glucose-regulated protein 78; LDHA, Lactate dehydrogenase A.

**Table 2:** Translational relevance and validation status of acylation-related candidates in UC.

Acylation Type	Candidate Biomarker or Axis	Clinical Association	Validation Status
Lactylation	H3K18la; circXRN2-*LATS1*-Hippo; *HNRNPA1*-K350la; *PRKN*-mediated mitophagy	M2 macrophage infiltration, tumor progression, immune evasion, possible prognosis association	UC-specific evidence is emerging; prospective clinical validation is lacking
Succinylation	*SIRT7*-*EZH2*-*RND3*; *LDHA* K222su as extrapolated model	Cisplatin resistance and metabolic adaptation	Mechanistic UC evidence exists for *SIRT7*/*EZH2*, but UC succinylome-wide clinical validation is lacking
Crotonylation (Kcr)	KAT2A-related pathways	Metastasis and poor survival in HCC; hypothetical relevance to UC signaling	No direct UC crotonylation biomarker validation identified
Palmitoylation	*MPP1*/*USP12*/*CCL5*; *ZDHHC9*/BiP; cisplatin-resistance-associated S-palmitoylation; B7-H4/*ZDHHC3* as extrapolated model	CD8^+^ T-cell infiltration, immune checkpoint response, progression, and drug resistance	UC-specific support is strongest for MPP1, *ZDHHC9*/BiP, and cisplatin-resistance-associated palmitoylation

Abb: UC, urothelial carcinoma; H3K18la, histone H3 lysine 18 lactylation; K350la, lysine 350 lactylation; K222su, lysine 222 succinylation; Kcr, lysine crotonylation; HCC, hepatocellular carcinoma; BiP, binding immunoglobulin protein; CD8, cluster of differentiation 8.

## 3 Mechanisms of Lactylation in UC

### 3.1 Biochemical Features and Enzymatic Control

Lactylation is a metabolism-sensitive PTM. It is characterized by the covalent addition of a lactyl group to lysine residues on histone or non-histone proteins. Lactylation was first identified as a histone lysine modification that links lactate accumulation with transcriptional regulation. Mechanistically, lactylation can occur through at least two biochemical routes. The first route depends on lactyl-CoA, which serves as an activated lactyl donor for enzyme-mediated lysine lactylation. The second route involves S-D-lactoylglutathione, an intermediate of the methylglyoxal pathway. This molecule may participate in non-enzymatic lysine lactylation under conditions of altered glycolytic flux and increased carbonyl stress [9,13]. These two routes suggest that lactylation is not only a passive consequence of lactate accumulation. Instead, it is a metabolism-regulated modification that can dynamically respond to glycolysis, hypoxia, mitochondrial activity, and cellular stress [21,22].

The enzymatic regulation of lactylation can be understood in terms of writers, erasers, and potential reader proteins. The p300/CBP family was among the first enzyme systems proposed to participate in histone lactylation. This supports the idea that some acyltransferases can use not only acetyl-CoA but also other acyl-CoA donors. Recent studies have expanded this view and identified additional regulatory mechanisms of lactylation, including the ACSS2-KAT2A axis, GTPSCS/SUCLG2-related lactyl-CoA generation, and non-histone lactylation [18,22]. However, these mechanisms have mainly been characterized in non-UC models. Their relevance to UC remains hypothetical and requires direct validation in UC tissues, organoids, or immune co-culture systems.

Lactylation is reversible, but the identity and specificity of delactylases remain incompletely defined. Histone deacetylases (HDACs) and members of the sirtuin family are considered possible enzymes that remove lactylation marks in different biological contexts [16]. For example, SIRT3-mediated delactylation of malic enzyme 2 (ME2) has been shown to affect redox balance and tumor-cell metabolism. This supports the functional importance of non-histone delactylation [23]. In prokaryotic systems, CobB has also been reported to remove lysine lactylation and regulate bacterial glycolysis. This suggests that the dynamic regulation of lactylation may be partly evolutionarily conserved. Compared with acetylation, the substrate specificity, catalytic efficiency, and cellular-context dependence of lactylation erasers remain insufficiently understood.

Lactylation can occur on both histone and non-histone proteins, but these two types of modification should be understood separately. In bladder cancer, direct evidence indicates that histone lactylation contributes to tumor progression. CircXRN2 suppresses bladder cancer progression by stabilizing LATS1 and activating the Hippo pathway. This reduces glycolysis-derived lactate production and decreases H3K18 lactylation. Mechanistically, reduced H3K18 lactylation downregulates LCN2 expression, thereby inhibiting malignant phenotypes [13]. This study provides one of the earliest direct links between lactylation and bladder cancer. It also suggests that lactylation can connect non-coding RNA regulation, Hippo signaling, and metabolic epigenetic control.

Non-histone lactylation is also considered an important mechanism in bladder cancer. Wang et al. showed that lactate-driven lactylation of HNRNPA1 promotes PKM2 splicing and glycolytic reprogramming in bladder cancer. In this study, p300-mediated lactylation of HNRNPA1 at lysine 350 enhanced its ability to regulate PKM pre-mRNA splicing. This favored PKM2 expression and strengthened the Warburg phenotype [14]. This finding is important because it shows that lactylation is not limited to chromatin regulation. It can also modify RNA-binding proteins and reshape cancer metabolism at the post-transcriptional level.

### 3.2 Lactylation-Driven Metabolic Reprogramming

Lactylation is currently the emerging acylation modification with the most direct support in UC, especially bladder UC. Since As a lactate-derived epigenetic modification, histone lysine lactylation links cellular metabolism with gene transcription, and increasing evidence suggests that it may connect glycolytic reprogramming, chromatin remodeling, immune regulation, and tumor progression [24]. This mechanism is particularly relevant to UC, because bladder cancer often shows enhanced glycolysis, hypoxia, lactate accumulation, and metabolic heterogeneity [25,26].

Evidence from non-UC cancer models suggests that lactylation may regulate glycolysis, mitochondrial metabolism, and redox balance through substrates such as PFKP or ME2 [23,27]. However, direct evidence that these mechanisms drive metabolic reprogramming in UC remains lacking. In bladder cancer, stronger support currently comes from UC-specific studies showing that H3K18 lactylation and HNRNPA1 lactylation are linked to glycolytic regulation, PKM2 splicing, and malignant phenotypes [13,14]. Therefore, PFKP- and ME2-related mechanisms should be regarded as hypotheses for UC rather than established UC pathways (Fig. 1).

**Figure 1:**
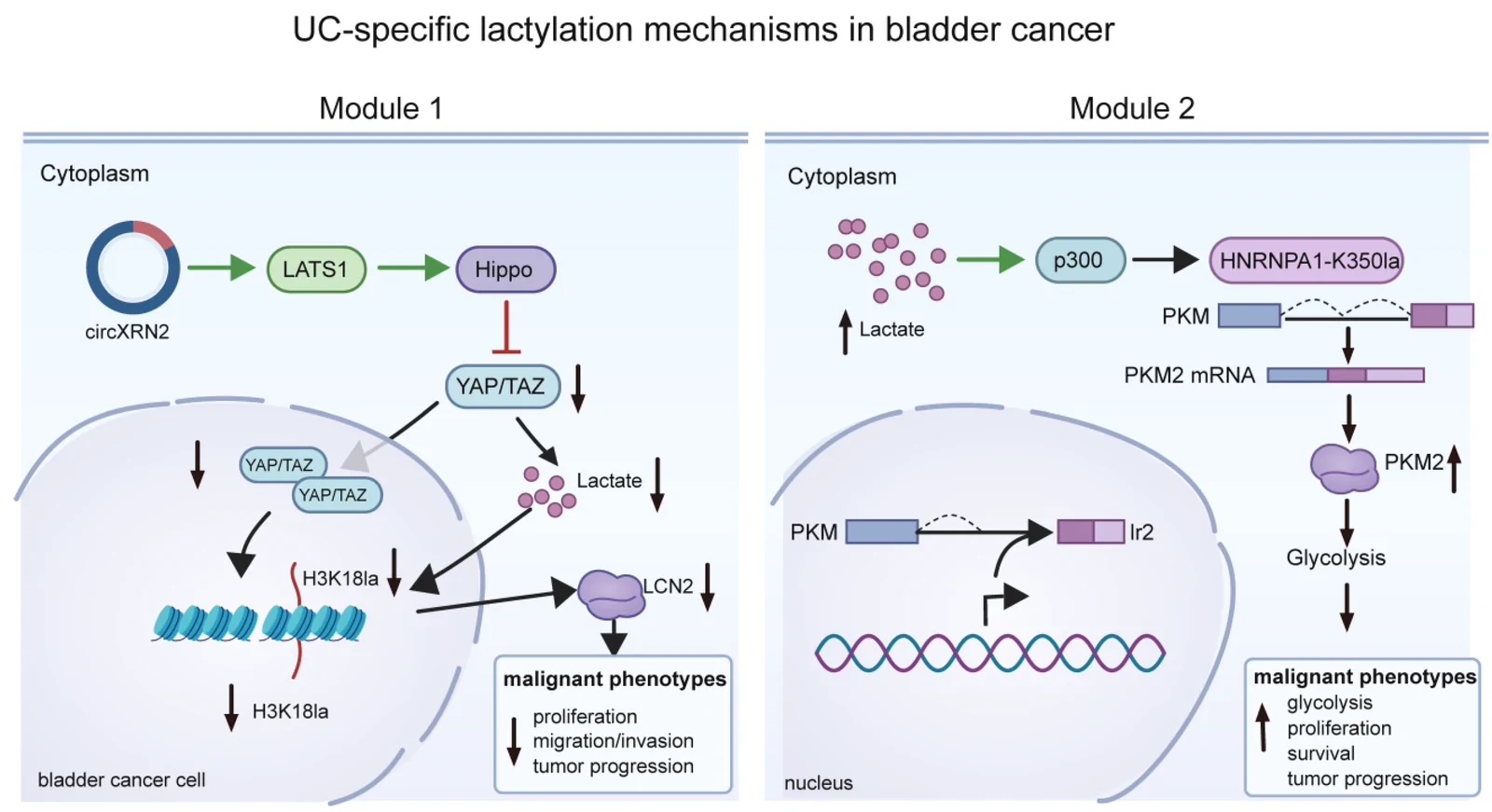
Urothelial carcinoma (UC)-specific lactylation mechanisms in bladder cancer. Module 1 illustrates the circXRN2–LATS1–Hippo signaling axis, in which stabilization of *LATS1* suppresses YAP/TAZ activity, reduces lactate production and H3K18 lactylation, and inhibits *LCN2*-associated malignant phenotypes. Module 2 shows that lactate-derived lactylation promotes *HNRNPA1*-K350 lactylation, which regulates PKM splicing and increases PKM2 expression, thereby enhancing glycolysis, proliferation, survival, and tumor progression. The figure was created using BioRender. Abb: UC, urothelial carcinoma; YAP/TAZ, Yes-associated protein/transcriptional coactivator with PDZ-binding motif; H3K18la, histone H3 lysine 18 lactylation; PKM, pyruvate kinase M; PKM2, pyruvate kinase M2; mRNA, messenger RNA.

### 3.3 Lactylation and the Tumor Immune Microenvironment

Cancer cells will undergo metabolic changes, leading to a large accumulation of lactic acid. These lactic acids are not only metabolic wastes, but also can add lactylation modification to histones and non-histones, affecting gene expression and cell activities. Lactylation can also change the way immune cells enter tumors, thus shaping the immune environment around tumors. It is found that the activity of lactylation-related genes is related to the number of macrophages, regulatory T cells (Tregs) and CD8^+^ T cells in many cancers.

In non-UC tumor models, lactate-driven lactylation has been linked to tumor immune regulation and the formation of an immunosuppressive microenvironment [28]. This mechanism provides a useful immune-regulatory model, but its direct relevance to UC remains to be tested. In bladder cancer, more direct evidence shows that lactylation promotes tumor-associated macrophages toward an M2-like state through PRKN-mediated mitophagy, thereby strengthening the immunosuppressive TME [15]. Other mechanisms, such as ACSS2-KAT2A-mediated histone lactylation or APOC2 lactylation-associated immunotherapy resistance, have been reported mainly in non-UC contexts and should be interpreted as hypothesis-generating examples for UC [18,29].

Single-cell transcriptomic analysis suggests an association between histone lactylation and cisplatin resistance in patients with bladder cancer. In addition, lactylation-related prognostic signatures and immunometabolic regulatory models have been established in bladder cancer. These findings suggest a potential link between lactylation, the tumor immune microenvironment, and immunotherapy response [30,31]. However, many of these prognostic studies are mainly based on transcriptomic inference rather than direct lactylome analysis. Therefore, their conclusions should be interpreted with caution.

For upper tract UC, including renal pelvic and ureteral UC, direct studies on lactylation remain limited. Similarly, urethral UC is rare, and dedicated mechanistic studies centered on lactylation are still lacking. Therefore, current evidence should not be generalized from bladder cancer to all UC subtypes. A more accurate conclusion is that lactylation is an emerging and experimentally supported mechanism in bladder UC, whereas its role in renal pelvic, ureteral, and urethral UC remains an important research gap.

## 4 Succinylation and Its Association with UC

### 4.1 Molecular Mechanisms and Biological Functions of Succinylation

Succinylation plays various important roles in cancer research. This biological process is about a chemical group called succinyl (-CO-CH_2_-CH_2_-CO-) which is covalently linked to a specific lysine amino acid in protein. Compared with acetylation, this modification changes the local charge of the protein from positive to negative. As a result, it can markedly alter protein conformation, enzymatic activity, stability, and protein interactions. Succinylation can occur in two ways. One route requires enzymatic catalysis, while the other does not require enzymes. In the enzymatic process, specific enzymes, such as lysine succinyltransferases, catalyze the transfer of the succinyl group from molecules such as succinyl-CoA to target lysine residues. In contrast, desuccinylases, such as SIRT5, remove this modification and maintain a stable balance of succinylation status [32,33].

Although succinylation of metabolic enzymes such as LDHA or SUCLG1 has been described in non-UC cancer or non-cancer models, UC-specific succinylome data remain limited [19,34,35]. Therefore, these examples are used here mainly to illustrate how succinylation may connect mitochondrial metabolism with cancer biology.

### 4.2 Roles in Proliferation and Migration

PI3K/AKT and MAPK/ERK pathways are critical for controlling the growth, survival and movement of UC cells. Current evidence suggests that succinylation-related metabolic regulation may crosstalk with these oncogenic signaling networks. For example, MLN4924, an inhibitor of NEDD8 activator, will disrupt the NEDD process, which in turn may influence the signal transmission related to succinylation. Interestingly, using MLN4924 alone unexpectedly activates PI3K/AKT and MAPK/ERK pathways, thus promoting cell survival and migration. However, when MLN4924 is combined with the selective COX-2 inhibitor celecoxib, it can effectively inhibit these pathways, inhibit the proliferation, migration and invasion of UC cells, promote cell death, and prevent epithelial-mesenchymal transition (EMT) [36]. However, this study did not directly map succinylated substrates or prove that succinylation controls AKT/ERK signaling in UC. Therefore, it should be interpreted as UC-related signaling evidence.

More direct evidence linking desuccinylation regulation with bladder cancer biology comes from studies on SIRT7 and EZH2. SIRT7 is a sirtuin mainly localized in the nucleus. It has deacetylase- and desuccinylase-related activities and is involved in ribosome biogenesis, chromatin regulation, stress responses, and cancer progression. In bladder cancer cells, SIRT7 has been reported to interact with EZH2 and regulate its succinylation status. This affects EZH2 stability, promoter-binding ability, RND3 repression, and cisplatin resistance. These findings suggest that succinylation-related regulation may contribute to chemotherapy tolerance in UC by affecting epigenetic regulators and their downstream transcriptional programs [37].

However, the exact succinylation sites, enzyme specificity, and broader substrate network involved in this process still need to be systematically mapped. Overall, succinylation may participate in UC progression by regulating mitochondrial metabolism, redox balance, metabolic enzyme activity, protein stability, and treatment-related signaling pathways. In UC, succinylation may be associated with metabolic plasticity, oxidative stress adaptation, and treatment tolerance. However, many mechanisms still require direct validation in UC-specific tissues, cell lines, organoids, and animal models.

### 4.3 Succinylation and Tumor Immune Regulation

Regarding immune regulation, succinylation may influence tumor immunity by modulating metabolic enzymes, chemokine production, and immune-cell function. A relevant finding is the tumor-suppressive role of the circSIRT5 axis in bladder cancer. Li et al. reported that circSIRT5 suppresses bladder cancer progression by promoting ferroptosis through the SYVN1/PHGDH pathway [38]. This study did not prove that lysine succinylation directly drives bladder cancer. However, it supports the importance of SIRT5-related metabolic regulation in bladder cancer biology. Because SIRT5 is a major desuccinylase, future studies should determine whether circSIRT5-related phenotypes are accompanied by changes in protein succinylation. Sirtuin deregulation may also influence bladder cancer invasion and immune-related phenotypes through epigenetic and metabolic crosstalk. Previous evidence showed that sirtuins are globally deregulated in bladder cancer, and SIRT7 downregulation is associated with epithelial-mesenchymal transition and increased invasiveness through the EZH2-CDH1 axis [39]. Together with the SIRT7/EZH2 succinylation findings, these results suggest that sirtuin-related acylation regulation may connect metabolic stress, chromatin remodeling, treatment resistance, and aggressive bladder cancer behavior.

Recent transcriptomic studies further suggest a potential association between succinylation-related genes, immune escape, and the TME in bladder cancer. A succinylation-related prognostic model was reported to predict patient outcomes and reveal links with immune escape, TME remodeling, and potential immunotherapy response [40]. However, these findings are mainly based on bioinformatic inference rather than direct succinylome profiling. Overall, succinylation-related regulation may influence the UC TME by modulating metabolic enzyme activity, chemokine signaling, immune-cell metabolism, and immunosuppressive pathways. Further studies using UC-specific tissues, single-cell analysis, succinyl-proteomics, and functional validation are required to determine whether succinylation directly regulates immune remodeling and immunotherapy response in UC.

## 5 Advances in Crotonylation Research in UC

### 5.1 Biology and Enzymology of Crotonylation

Compared with acetylation, the crotonyl group contains a planar carbon-carbon double bond. Therefore, it has unique spatial and electronic properties. These features may affect protein charge, conformation, molecular recognition, and protein interactions. As a result, crotonylation may participate in the regulation of gene expression, metabolic control, DNA repair, and cell-cycle regulation [41,42]. Direct UC-specific evidence for crotonylation remains scarce. Therefore, this section first summarizes the basic biology of crotonylation from broader cancer and non-cancer studies, and then discusses its possible relevance to UC as a hypothesis-generating direction.

Crotonylation is currently best defined as a histone lysine acylation mark associated with active transcription. The unique structure of the crotonyl group may weaken histone-DNA interactions and promote the recruitment of specific regulatory proteins. In this way, crotonylation contributes to chromatin relaxation and transcriptional activation [41,42]. In cancer cells, crotonylation can adjust the activity of metabolic enzymes and change the process of energy production and cell cycle, thus affecting the growth and spread of cancer cells [41,42,43,44].

The metabolic donor for crotonylation, crotonyl-CoA, can be derived from fatty acid metabolism, amino acid catabolism, and short-chain acyl-CoA metabolic pathways. Therefore, crotonylation may act as a molecular bridge between cellular metabolic status and protein regulation. The crosstalk between crotonylation and acetylation is particularly important. Both modifications occur on lysine residues and may share writers, erasers, or nearby substrate sites. Changes in the relative abundance of acetyl-CoA and crotonyl-CoA may affect the balance between histone acetylation and crotonylation, thereby altering transcriptional output. However, the substrate specificity of crotonylation-related enzymes and the determinants of site-selective crotonylation remain incompletely defined.

Crotonylation is also involved in immune cell activity and inflammatory reaction, so it is also an important direction in immune metabolism research.

### 5.2 Crotonylation in Oncogenic Signaling

The PI3K/AKT and MAPK/ERK signaling pathways are core pathways in UC. However, current evidence is insufficient to show that crotonylation directly regulates these pathways in UC. Studies from non-UC models suggest that crotonylation may influence protein function, metabolic adaptation, and stress-related signaling. These findings support the general concept that crotonylation can regulate cancer-related cellular processes [41,45].

In cancer, crotonylation may regulate both histone and non-histone proteins. Histone crotonylation is associated with transcriptional activation. Non-histone crotonylation may affect protein stability, enzyme activity, ubiquitination, and signal transduction. Studies in non-UC models suggest that crotonylation may influence metabolic enzyme activity and cancer-related signaling pathways. This study provides a useful model showing how site-specific non-histone crotonylation can regulate oncogenic signaling pathways. In UC, crotonylation should therefore be regarded as a promising research direction rather than an established mechanism.

Future studies in UC should prioritize the identification of crotonylated substrates and functional sites in UC tissues and models.

### 5.3 Crosstalk with TME

Crotonylation may be related to tumor microenvironmental regulation, particularly through effects on inflammatory signaling, immune-cell states, and cancer-associated fibroblasts. However, most available evidence comes from non-UC cancers. In hepatocellular carcinoma and glioma, crotonylation-related signatures have been associated with Tregs, TAMs, and immune checkpoint expression, including PD-1/PD-L1 [46,47]. These observations provide a rationale for investigating crotonylation in the UC immune microenvironment, but they do not prove a direct role for crotonylation in UC immune escape.

In several cancer types, crotonylation-related gene signatures are associated with immune-cell infiltration, inflammatory signaling, and immune checkpoint expression. For example, studies in hepatocellular carcinoma and glioma suggest that increased crotonylation-related activity may be linked to higher levels of regulatory T cells, tumor-associated macrophages, and PD-1/PD-L1 expression. These findings suggest a possible connection between crotonylation and immune remodeling. Crotonylation may be particularly relevant to transcriptional plasticity in UC. Bladder cancer often undergoes molecular subtype switching, epithelial-mesenchymal transition, therapy-induced transcriptional adaptation, and remodeling of the immune microenvironment [48,49]. Because histone crotonylation is generally associated with active transcription, altered crotonylation may contribute to oncogene expression, lineage plasticity, DNA damage responses, or immune escape. However, these mechanisms remain hypothetical in UC and require direct experimental validation.

Overall, crotonylation represents an emerging direction in acylation regulation. It may be related to metabolism, signal transduction, and immune regulation in UC. At present, the main value of crotonylation is to generate mechanistic hypotheses and provide new directions for acylomics-based biomarker discovery. To establish crotonylation as a true regulatory mechanism in UC, future studies need to define UC-specific crotonylation substrates, validate site-specific functions, identify related writers and erasers, and analyze the relationships between crotonylation features and clinical stage, molecular subtype, immune infiltration, treatment response, and patient prognosis.

## 6 Palmitoylation in UC: Roles and Clinical Significance

### 6.1 Biological Basis and Regulatory Mechanisms

Palmitoylation is mechanistically distinct from lactylation, succinylation, and crotonylation. The latter three mainly modify lysine residues and are often related to chromatin regulation or metabolic enzyme control. In contrast, palmitoylation usually refers to reversible S-palmitoylation of cysteine residues. This lipid modification increases protein hydrophobicity and regulates membrane localization, vesicle trafficking, receptor stability, and signal transduction [50,51,52].

Palmitoylation is mainly catalyzed by palmitoyl acyltransferases that contain a zinc-finger Asp-His-His-Cys (zDHHC) domain. These enzymes recognize specific cysteine motifs or structural contexts in target proteins and transfer the palmitoyl group from palmitoyl-CoA to substrate proteins [53,54]. Different zDHHC enzymes have distinct subcellular localization and substrate preferences. For example, zDHHC5 and zDHHC8 are enriched in neuronal axons and participate in axonal signaling and transport. zDHHC17 has been reported to palmitoylate AMPKα and regulate energy metabolism [53]. Palmitoylation is also reversible and can be removed by depalmitoylases such as acyl-protein thioesterases and ABHD family members. Therefore, the palmitoylation status of a protein is jointly determined by palmitoyl-CoA availability, zDHHC enzyme activity, depalmitoylase activity, substrate accessibility, and cellular localization.

In cancer, imbalance of zDHHC enzymes and abnormal palmitoylation may alter oncogenic signaling, metabolic adaptation, membrane receptor function, and immune regulation. These mechanisms may provide effective therapeutic approaches for UC. For example, DHHC9 and DHHC15 are upregulated in some malignancies and are associated with increased cancer cell invasiveness [55,56,57]. Some compounds, such as 2-bromopalmitate, which are specially used to block palmitoylating enzyme or regulate palmitoylation, have been shown to inhibit the proliferation and growth of tumor cells in different experimental systems [58,59]. But broad palmitoylation inhibitors limited selectivity and potential off-target effects restrict direct clinical translation. In UC, future therapeutic studies should focus on disease-relevant enzyme-substrate pairs rather than global palmitoylation inhibition.

### 6.2 Control of UC Cell Proliferation and Migration

Palmitoylation plays an important role in regulating the localization, stability, and signaling activity of membrane-associated proteins. In UC, MPP1, a membrane-associated palmitoylated protein, has been implicated in tumor progression and immune remodeling. MPP1 is downregulated in UC, whereas higher MPP1 expression is associated with better patient prognosis and stronger immune infiltration. Functional experiments suggest that MPP1 overexpression inhibits the proliferation and migration of bladder cancer cells, indicating that MPP1 may have a tumor-suppressive role in UC [16].

Functionally, MPP1 can regulate the signal pathway and the recruitment of immune cells. According to the analysis of TCGA and BEST databases, the survival rate of patients and their response to immune checkpoint inhibition are related to the level of MPP1. Experimental data further suggest that MPP1 can enhance USP12 activity and promote CCL5 expression, thereby increasing CD8^+^ T-cell recruitment and reducing immune escape. In addition, MPP1 can stimulate the secretion of CCL5, which is a chemokine that can attract CD8^+^ T cells and help them penetrate, thus preventing tumors from escaping from the immune system. This regulation process depends on the interaction between MPP1 and ubiquitination enzyme USP12. MPP1 increased the activity of USP12 and the expression of CCL5, forming an MPP1/USP12/CCL5 axis, remodeling the immune environment of tumor and inhibiting the development of cancer [16]. On the other hand, known oncoproteins need palmitoylation to locate on the cell membrane. RAS family members, such as NRAS and HRAS, rely on this lipid modification to activate RAF/MEK/ERK and PI3K/AKT signaling pathways [20,60,61].

However, whether zDHHC9-driven RAS palmitoylation is a major functional driver in UC still requires direct validation. Therefore, targeting MPP1-related immune regulation or abnormal RAS palmitoylation may represent a potential therapeutic direction, but further UC-specific mechanistic and preclinical studies are needed. More direct UC evidence comes from the ZDHHC9-BiP/GRP78 axis. ZDHHC9-mediated S-palmitoylation of BiP/GRP78 has been reported to inhibit unfolded protein response signaling and promote bladder cancer progression. This finding identifies a specific enzyme-substrate pair in bladder cancer and provides stronger UC-specific support than broad extrapolation from RAS palmitoylation models [17] (Fig. 2).

**Figure 2:**
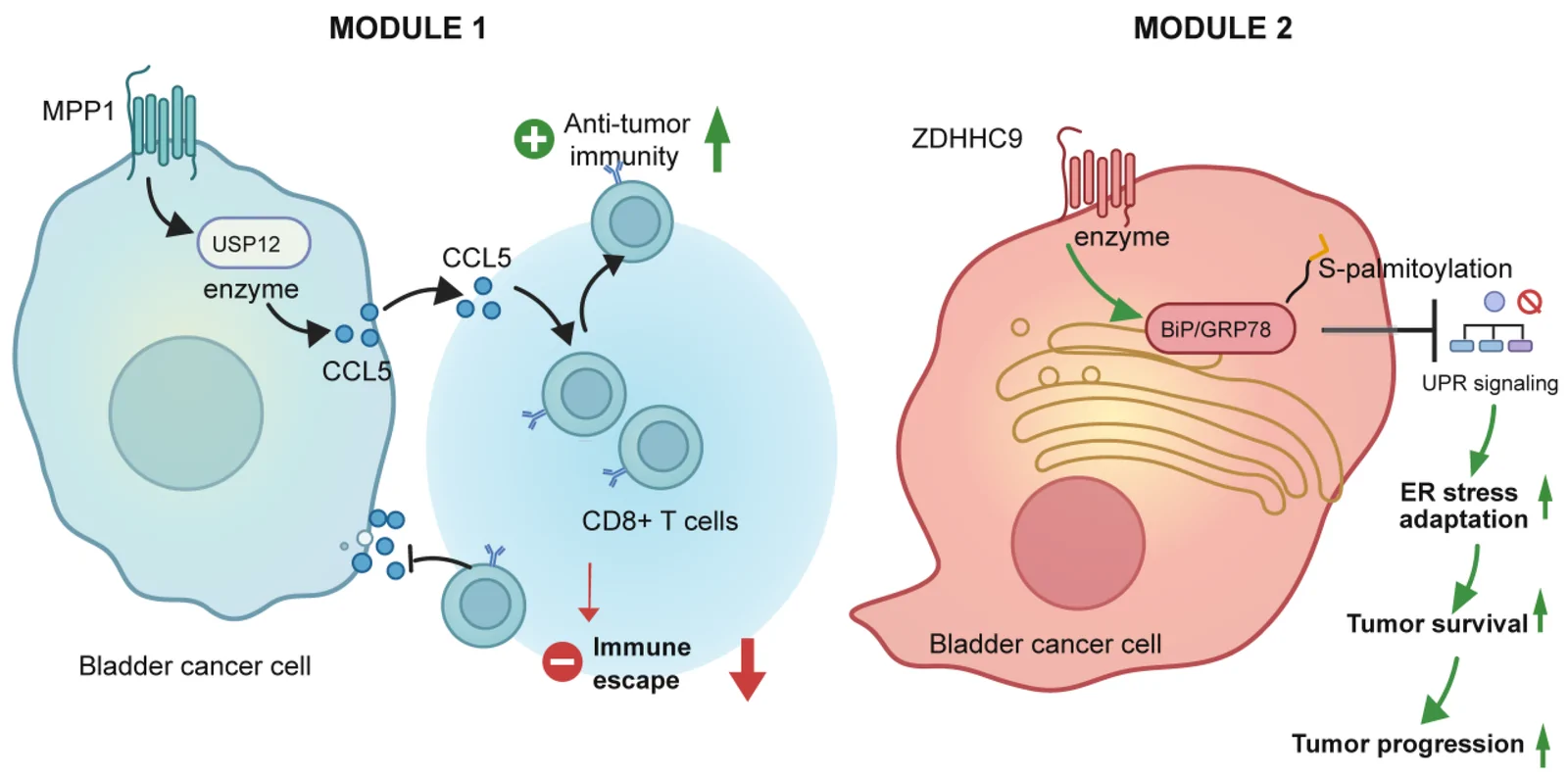
Palmitoylation-related mechanisms involved in immune evasion and tumor progression in bladder cancer. Module 1 illustrates the *MPP1*–*USP12*–*CCL5* axis in bladder cancer cells, in which MPP1-associated signaling promotes CCL5-mediated CD8^+^ T-cell recruitment and enhances anti-tumor immunity, thereby reducing immune escape. Module 2 shows that *ZDHHC9*-mediated S-palmitoylation of BiP/GRP78 contributes to unfolded protein response signaling, endoplasmic reticulum stress adaptation, tumor survival, and tumor progression. The figure was created using BioRender. Abb: CD8, cluster of differentiation 8; BiP/GRP78, binding immunoglobulin protein/glucose-regulated protein 78; UPR, unfolded protein response; ER, endoplasmic reticulum.

### 6.3 Immune Evasion and Immunotherapy Potential

One potentially important mechanism is immune checkpoint regulation. Yao et al. reported that PD-L1 palmitoylation stabilizes PD-L1 and promotes tumor immune evasion, while inhibition of PD-L1 palmitoylation enhances antitumor T-cell responses [62]. Since PD-1/PD-L1 immune checkpoint blockade has major clinical relevance in advanced UC, palmitoylation-dependent stabilization of PD-L1 may represent a plausible mechanism of immune escape in UC. However, whether PD-L1 palmitoylation directly regulates immune evasion in bladder or upper tract UC still requires experimental confirmation.

Another relevant pathway is Hippo-YAP/TEAD signaling. TEAD transcription factors require palmitoylation for proper folding, stability, and transcriptional function [63]. YAP/TEAD signaling is associated with bladder cancer progression, epithelial-mesenchymal transition, stemness, and treatment resistance [64,65]. Therefore, TEAD palmitoylation may indirectly influence UC by supporting YAP/TEAD-dependent transcriptional programs. This is a strong mechanistic hypothesis, but direct evidence in UC is still lacking.

Palmitoylation may also be related to FGFR3-driven UC. FGFR3 alterations are common in non-muscle-invasive bladder cancer and upper tract UC, and FGFR-targeted therapy has become an important treatment option for some patients with advanced UC [66,67]. Although the direct role of FGFR3 palmitoylation in UC remains unclear, palmitoylation can regulate membrane-associated signaling proteins and receptor trafficking [50,51,52]. Therefore, in FGFR3-altered UC, altered ZDHHC enzyme activity may potentially affect receptor localization, signaling duration, or drug response.

Overall, palmitoylation should be regarded as an emerging and mechanistically plausible acylation-related modification in UC, rather than an established UC marker. Its potential relevance mainly lies in membrane signaling, PD-L1 stability, YAP/TEAD activity, FGFR3/EGFR pathway regulation, and immune escape. Future studies should use acyl-biotin exchange, acyl-resin-assisted capture, and palmitoyl-proteomics to characterize palmitoylation in bladder and upper tract UC. Functional studies should prioritize ZDHHC enzymes, PD-L1, TEADs, FGFR3, EGFR, and RAS family proteins.

## 7 Discussion

Protein acylation provides a metabolism-sensitive regulatory layer. It may link metabolic reprogramming in UC with chromatin regulation, mitochondrial function, membrane signaling, immune remodeling, and treatment response [30,68]. Unlike relatively stable genetic alterations, acylation modifications are dynamic. They can rapidly respond to changes in nutrient supply, hypoxia, redox status, and acyl-CoA donor pools. Metabolic intermediates can also be used as signal molecules to promote the acylation process, which provides multiple opportunities for clinical intervention.

Current evidence supports a central concept: lactylation, succinylation, crotonylation, and palmitoylation should not be viewed as isolated events. Instead, they may be better understood within a shared “metabolism-acylation” network. Enhanced glycolysis may increase the availability of lactate and lactyl-CoA, thereby supporting lactylation. Mitochondrial remodeling and tricarboxylic acid cycle activity may affect succinyl-CoA levels and succinylation. Fatty acid and amino acid metabolism may contribute to crotonyl-CoA production and influence crotonylation. Lipid metabolism provides palmitoyl-CoA for palmitoylation. These acyl donor pools are metabolically interconnected. They are also influenced by acetyl-CoA availability, NAD^+^-dependent sirtuin activity, and cellular stress states. Therefore, metabolic reprogramming in UC may reshape the global acylation landscape rather than affect only one specific modification.

Therefore, future studies should investigate acylation modifications in UC as an integrated metabolite-sensitive network rather than as four independent pathways.

Several important gaps remain. First, most UC studies evaluate acylation-related genes, enzymes, or global antibody signals rather than site-resolved modification events. As a result, it is often unclear which substrate, residue, cell type, or subcellular compartment is responsible for the observed phenotype. Second, bladder cancer evidence should not be automatically generalized to upper tract or urethral UC, where acylation-centered studies are still scarce. Third, many proposed biomarkers are based on transcriptomic inference, public-dataset modeling, or studies from other cancers. These approaches are useful for generating hypotheses, but they cannot replace direct lactylome, succinylome, crotonylome, or palmitoyl-proteome validation in UC tissues. Fourth, the same acylation-related pathway may have different effects depending on the substrate and context.

Future UC-specific studies should move beyond general references to CRISPR-Cas9, machine learning, or artificial intelligence. For lactylation, priority candidates include H3K18la, HNRNPA1-K350la, and enzymes involved in lactate-to-lactylation conversion. CRISPR-based perturbation, residue-specific mutagenesis, and rescue experiments could test whether these candidates regulate glycolytic reprogramming, LCN2 expression, macrophage polarization, cisplatin resistance, or immunotherapy response in bladder cancer cells, organoids, and tumor-immune co-culture systems [13,14,15]. For succinylation, the SIRT7/EZH2/RND3 axis should be prioritized because of its link with cisplatin resistance [37]. For crotonylation, the immediate priority is discovery-stage UC crotonylome profiling rather than direct clinical translation, because current mechanistic evidence is mainly derived from non-UC models [41,69]. For palmitoylation, cysteine-site mutagenesis and zDHHC perturbation should focus on RAS-family proteins, MPP1-related immune regulation, and cisplatin-resistance-associated S-palmitoylated proteins [16,59,60].

Machine-learning models should address specific UC clinical questions, such as whether H3K18la predicts immunosuppressive macrophage infiltration, whether MPP1/CCL5 predicts benefit from immune checkpoint blockade, whether SIRT7/EZH2-related features predict cisplatin resistance, and whether palmitoylation-associated signatures identify therapy-resistant tumors. These models should be trained and validated in independent UC cohorts and compared with standard clinicopathological predictors [16,30,31,37].

Combination strategies involving acylation-related pathways, chemotherapy, immunotherapy, targeted therapy, or metabolic inhibitors may be conceptually attractive, but they remain speculative and require rigorous UC-specific preclinical validation.

Several challenges must be addressed before acylation markers or therapeutic strategies can be incorporated into the clinical management of UC. First, many acylation regulators have broad substrate spectra. The same enzyme may regulate multiple substrates or several types of acylation. Second, acylation modifications are highly dynamic and context-dependent. Therefore, increased acylation levels may act as causal drivers of malignant progression, or they may simply represent adaptive responses to metabolic stress. Third, current detection methods, including mass spectrometry, acylation-specific antibodies, and metabolic labeling, all have limitations. These include low modification stoichiometry, antibody cross-reactivity, incomplete site coverage, and sample heterogeneity [70,71]. These limitations directly affect the interpretation of acylation data and highlight the need for cross-validation using multiple methods.

## 8 Conclusions

Protein acylation represents an emerging regulatory layer linking metabolism, epigenetic control, mitochondrial function, membrane signaling, immune remodeling, and treatment response in UC. Current evidence is strongest for lactylation and palmitoylation-related mechanisms in bladder cancer, whereas succinylation and crotonylation remain less validated and largely hypothesis-generating. Future studies should prioritize UC-specific acylome profiling, site-level functional validation, and integration with clinical cohorts to determine whether these modifications are causal drivers, adaptive responses, or clinically useful biomarkers.

## Data Availability

Not applicable. No new datasets were generated or analysed in this review. All information is derived from previously published studies.
